# Supplementary material for: Solution-Processed Large-Area Organic/Inorganic Hybrid Antireflective Films for Perovskite Solar Cell
Source: Molecules. 2023 Feb 24;28(5):2145. doi: 10.3390/molecules28052145 (PMC10004043; doi:10.3390/molecules28052145)
Supplement: Supplementary file 1 [file molecules-28-02145-s001.zip › molecules-2204217-supplementary.pdf]

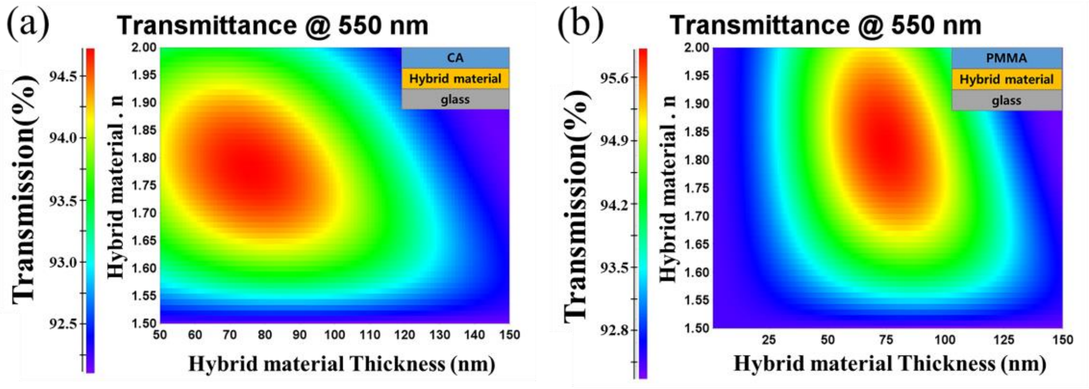

Figure S1 Fitting the optical structure using SetFos, the substrate refractive index is set to n_{sub}=1.5045. Chromatograms of the variation of transmittance with refractive index and film thickness of the hybrid material for single-sided bilayer antireflection films (10×10 cm^2): (a) hybrid material /CA structure; (b) hybrid material /PMMA structure.

Table S1 Effect of viscosity of PT solution of hybrid materials with aging time. (Storage temperature 5°C, test temperature 25°C)

Time (day)	0	7	14	30	60	90	120	180	240
Viscosity (mPa · s)	1.4	1.41	1.44	1.48	1.48	1.47	1.47	1.47	1.47

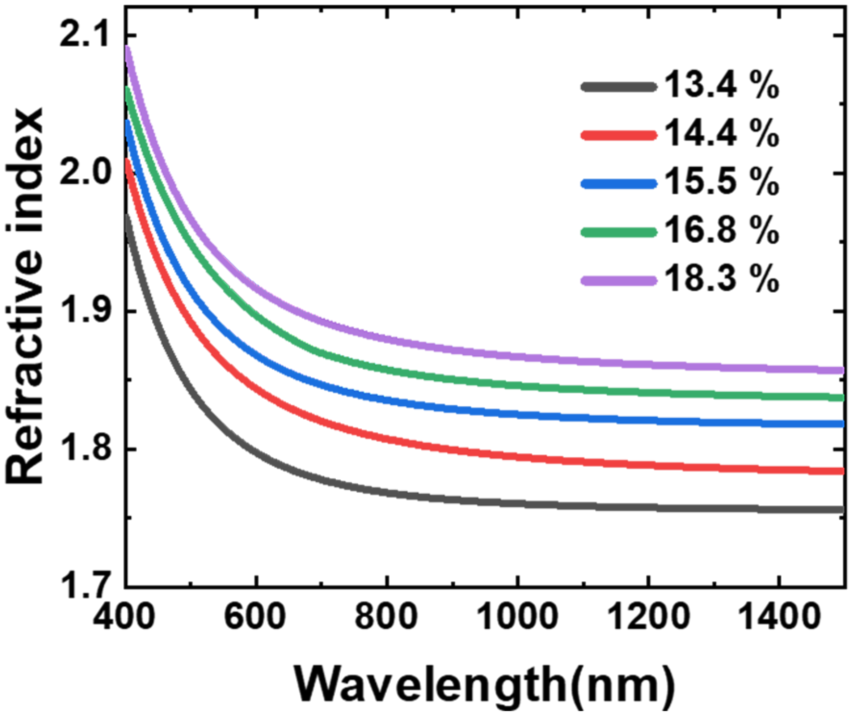

f

Figure S2 Refractive index spectra of different ratios of the hybrid material PT. The films were prepared at room temperature and annealed at 150 °C.

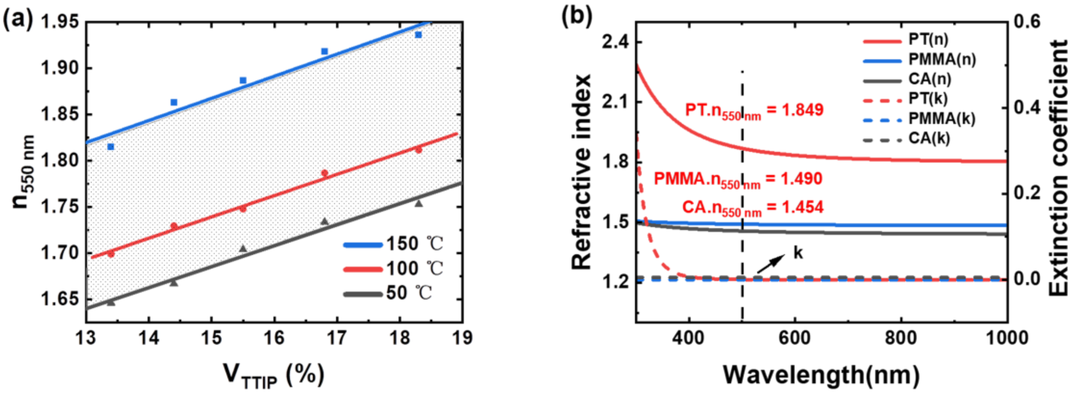

Figure S3 (a) Refractive index plots of different ratios of the hybrid material PT at different temperatures. (b) Ellipsometry measurement of refractive index and extinction coefficient of the hybrid material PT (14.4%) and polymer materials CA and PMMA, both prepared on silicon wafers. The refractive index was fitted using the Cauchy model with three Cauchy coefficients of 1.798, 3.14×10^3, and 3.72×10^9, respectively.

Table S2 Molar ratio of the materials in the hybrid material PT (14.4%) when 1 g of PVA was used.

Materials	TTIP	HAcAc	AcOH
Molar ratio	1	11	5.13

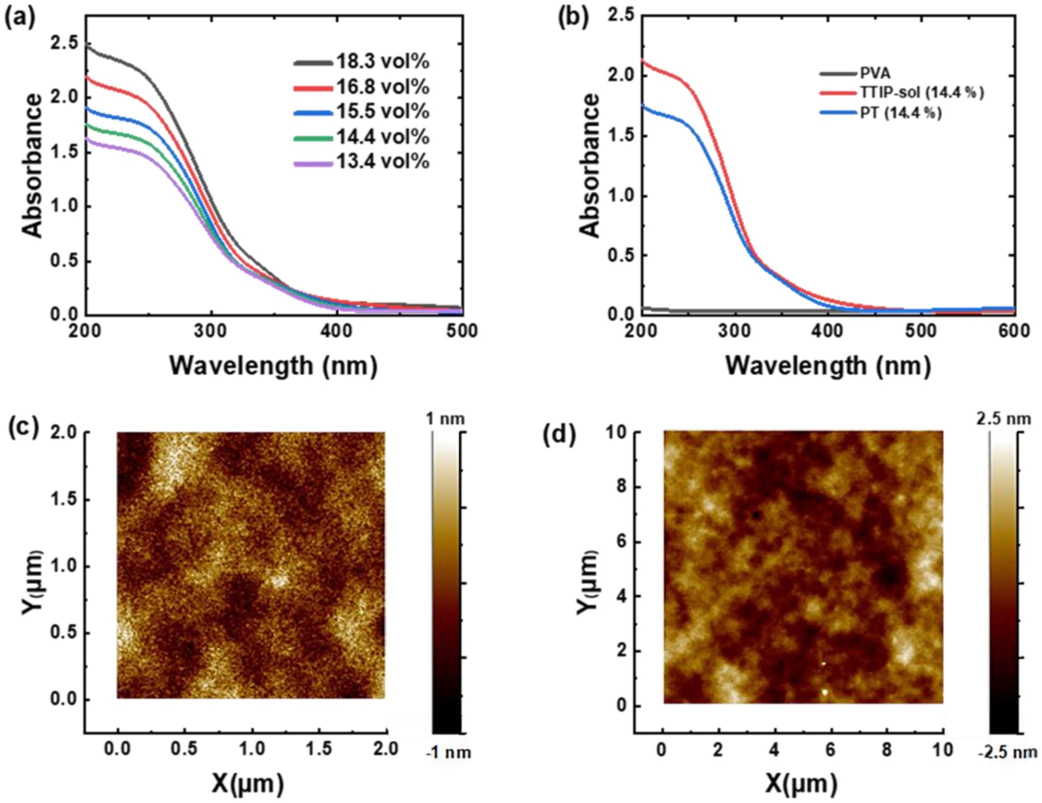

Figure S4 (a) Absorbance of PT films prepared on quartz substrates with different ratios of hybrid materials measured using UV spectrophotometer; (b) Absorbance of PVA, TTIP-sol, and PT film materials prepared on quartz substrates. (c) AFM of PT (14.4 %) films measured at a size of 2 μm × 2 μm. (d) AFM of PT (14.4 %) films measured at a large size of 10 μm × 10 μm. The annealing temperature was set at 150°C.

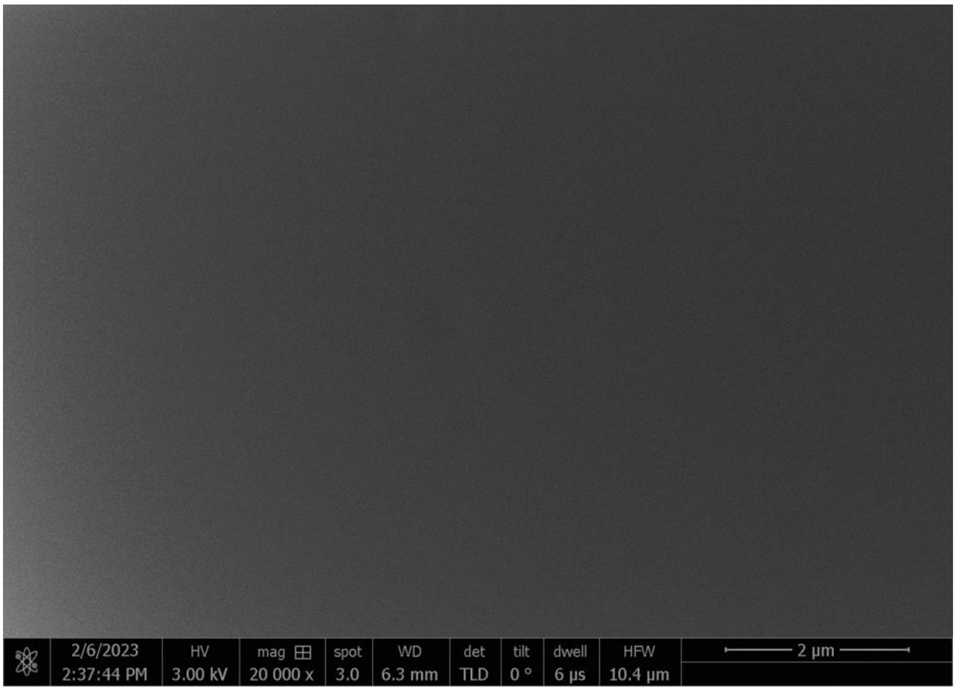

Figure S5 Scanning electron microscope (SEM) morphology of hybrid material PT (14.4%) films.

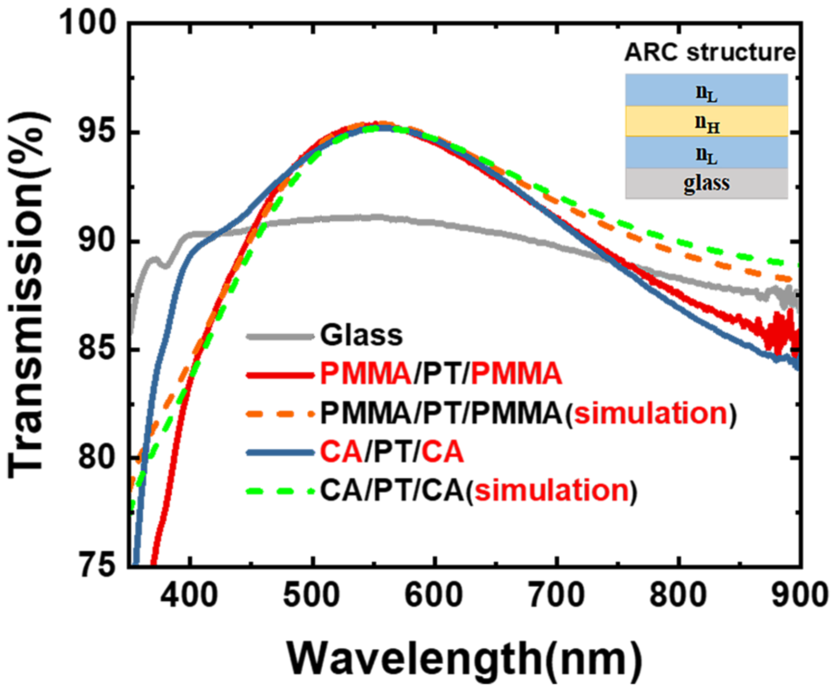

Figure S6 (a) Transmission spectra of triple-layer films with CA/PT/CA structure and PMMA/PT/PMMA structure. n_H: high refractive index hybrid material PT; n_L: low refractive index material CA and PMMA.

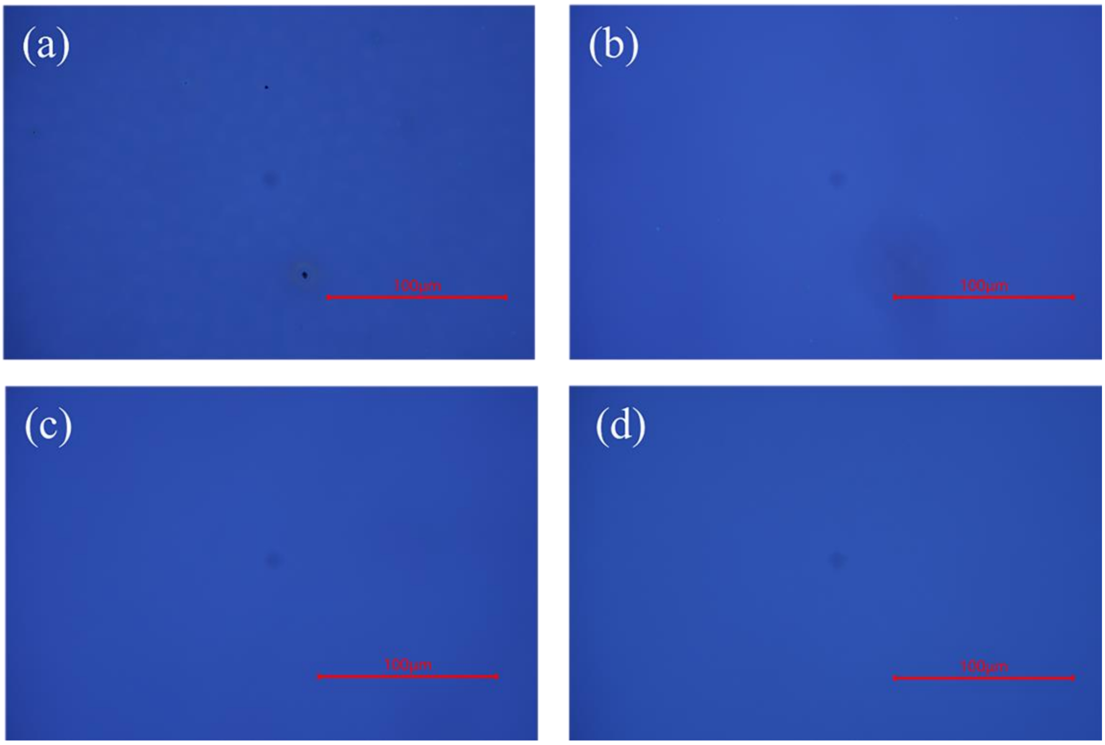

Fig. S7 Micrographs of the triple-layer structure of the antireflective films taken by an Olympus microscope. The antireflective films prepared by CA using acetonitrile (a), acetone (b), and DMAc (c) respectively as solvent to construct the CA/PT/CA structure. (d) PMMA/PT/PMMA film stacks.

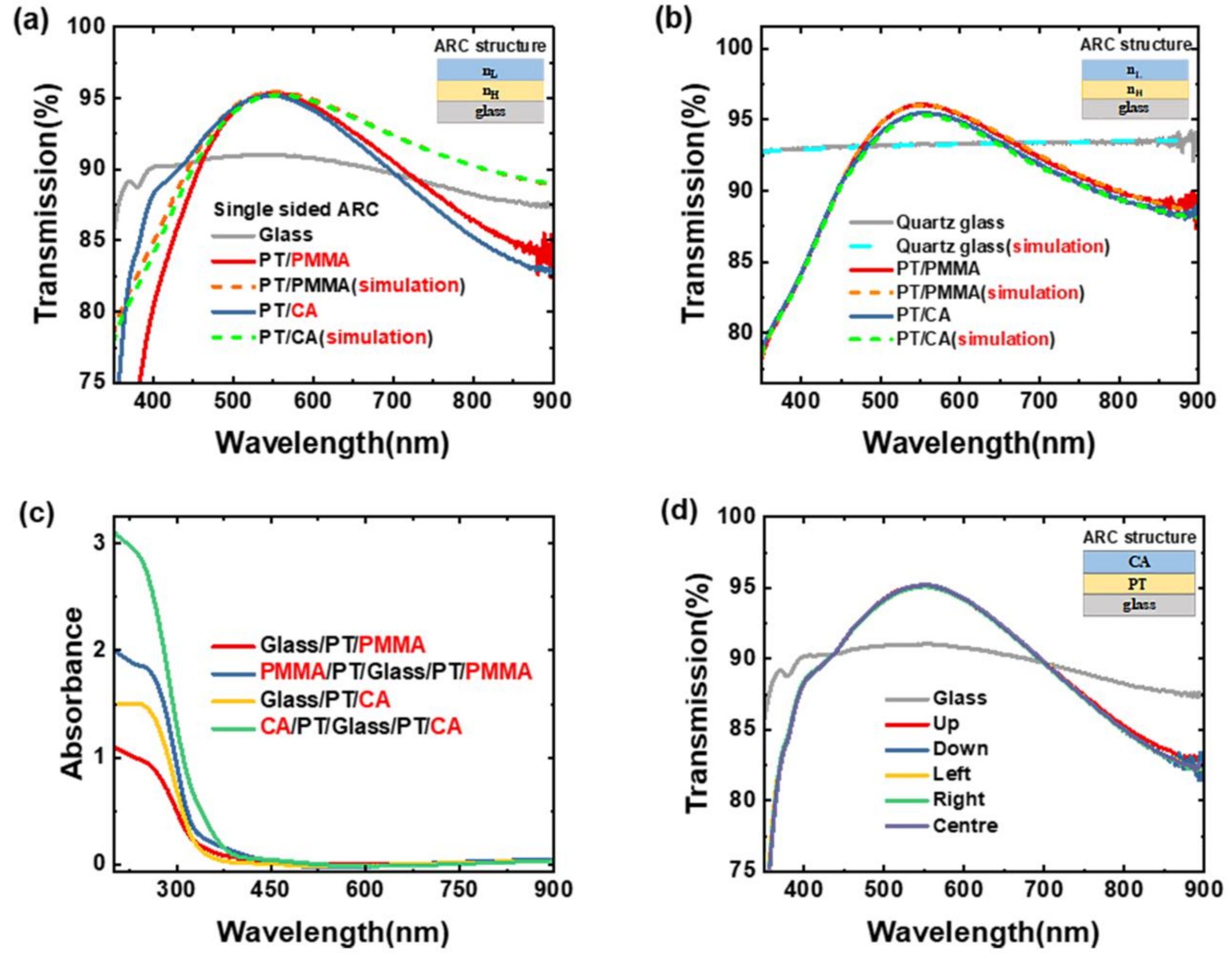

Figure S8 (a) Transmittance spectra of single-sided antireflective films of large-area PT/CA structure and PT/PMMA structure; (b) Transmission spectra of bilayer films with PT/CA structure and PT/PMMA structure. The refractive index and extinction coefficient of the simulated quartz substrate was set to 1.551 and 1.1×10^{-7}, respectively. (c) Absorption of different types of the single-sided and double-sided antireflective films. (d) Transmission spectra of the large-area and single-sided PT/CA antireflective films measured at five different positions: top, bottom, left, right, and center, respectively. n_H: high refractive index hybrid material PT. n_L: low refractive index material CA. The device structure of inverted perovskite solar cells is ITO/PTAA/Perovskite/PCBM/BCP/Ag [29]. The area of the active layer was 0.04 cm^2.

Table S3 Performance parameters of perovskite solar cell modules before and after preparation of PT/CA antireflective films for the cover glass (Device area: 0.04 cm^2)

Perovskite solar cells	J_{SC} (mA/cm^2)	J_{SC}(EQE) (mA/cm^2) ^{a}	FF (%)	V_{OC} (V)	PCE (%)
With cover glass	21.32	18.91	79.91	0.97	16.57
With single ARC (PT/PMMA)	21.48	19.73	79.57	0.99	16.95
With double sided ARC (PT/PMMA)	21.73	19.95	79.22	0.99	17.11

[a]Current desnity calculated from the EQE.The device structure of inverted perovskite solar cell is ITO/PTAA/Perovskite/PCBM/BCP/Ag [29]. The area of the active layer was 0.04 cm^2.

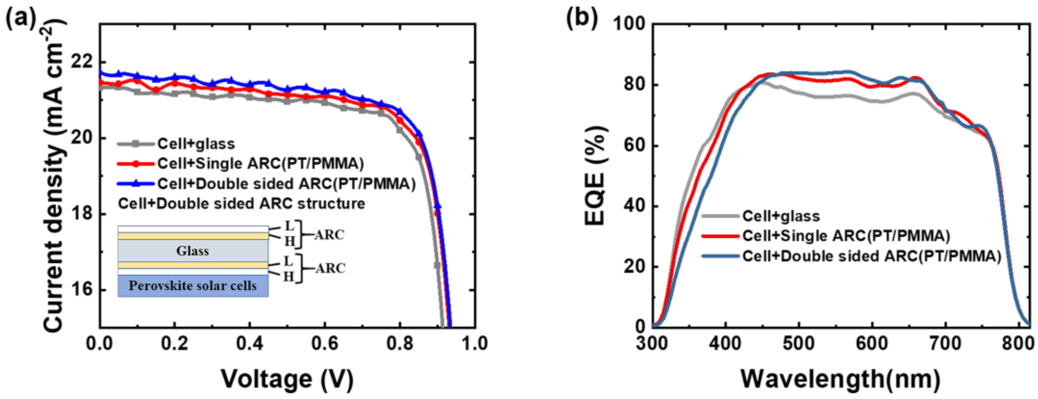

Figure S9 Performance parameters of perovskite solar cell modules with PT/PMMA structured single-sided and double-sided antireflective films prepared on cover glass. (a) J-V curves; (b) EQE plots. H: high refractive index hybrid material PT. L: low refractive index material PMMA.
